# DeepSynergy: predicting anti-cancer drug synergy with Deep Learning

**DOI:** 10.1093/bioinformatics/btx806

**Published:** 2017-12-15

**Authors:** Kristina Preuer, Richard P I Lewis, Sepp Hochreiter, Andreas Bender, Krishna C Bulusu, Günter Klambauer

**Affiliations:** 1 Institute of Bioinformatics, Johannes Kepler University, Linz, Austria; 2 Department of Chemistry, Centre for Molecular Science Informatics, University of Cambridge, Cambridge, UK; 3 Oncology Innovative Medicines and Early Development, AstraZeneca, Hodgkin Building, Chesterford Research Campus, Saffron Walden, Cambs, UK

## Abstract

**Motivation:**

While drug combination therapies are a well-established concept in cancer treatment, identifying novel synergistic combinations is challenging due to the size of combinatorial space. However, computational approaches have emerged as a time- and cost-efficient way to prioritize combinations to test, based on recently available large-scale combination screening data. Recently, Deep Learning has had an impact in many research areas by achieving new state-of-the-art model performance. However, Deep Learning has not yet been applied to drug synergy prediction, which is the approach we present here, termed DeepSynergy. DeepSynergy uses chemical and genomic information as input information, a normalization strategy to account for input data heterogeneity, and conical layers to model drug synergies.

**Results:**

DeepSynergy was compared to other machine learning methods such as Gradient Boosting Machines, Random Forests, Support Vector Machines and Elastic Nets on the largest publicly available synergy dataset with respect to mean squared error. DeepSynergy significantly outperformed the other methods with an improvement of 7.2% over the second best method at the prediction of novel drug combinations within the space of explored drugs and cell lines. At this task, the mean Pearson correlation coefficient between the measured and the predicted values of DeepSynergy was 0.73. Applying DeepSynergy for classification of these novel drug combinations resulted in a high predictive performance of an AUC of 0.90. Furthermore, we found that all compared methods exhibit low predictive performance when extrapolating to unexplored drugs or cell lines, which we suggest is due to limitations in the size and diversity of the dataset. We envision that DeepSynergy could be a valuable tool for selecting novel synergistic drug combinations.

**Availability and implementation:**

DeepSynergy is available via www.bioinf.jku.at/software/DeepSynergy.

**Supplementary information:**

Supplementary data are available at *Bioinformatics* online.

## 1 Introduction

Administering drug combinations instead of monotherapy can lead to an increased efficacy compared to single drug treatments (Csermely *et al.*, 2013; Jia *et al.*, 2009). Furthermore, host toxicity and adverse side effects are likely reduced, since doses of drug combinations are typically lower than doses of single agents (Chou, 2006; O’Neil *et al.*, 2016). Drug resistance can be decreased or even overcome through combination therapy (Huang *et al.*, 2016; Kruijtzer *et al.*, 2002; Tooker *et al.*, 2007). Therefore, drug combinations are investigated across various medical areas, such as cancer (Al-Lazikani *et al.*, 2012), viral disease including human immunodeficiency virus (HIV) and hepatitis C virus infections (Clercq, 2007) as well as fungal (Chen *et al.*, 2016; Groll and Tragiannidis, 2009) and bacterial infections (Tamma *et al.*, 2012; Worthington and Melander, 2013). However, drug combination effects can be adverse and even lead to shorter progression free survival of cancer patients (Hecht *et al.*, 2009; Tol *et al.*, 2009). Therefore, finding synergistic drug pairs for a particular cancer type is important for improving efficacy of anticancer treatment.

Until recently, effective drug combinations were proposed based on clinical experience. However, ‘trial-and-error’ is time-, labor- and cost-intensive. Furthermore, it might expose patients to unnecessary or even harmful treatment (Day and Siu, 2016; Pang *et al.*, 2014). Another strategy to identify synergistic drug pairs without harming patients is high-throughput screening (HTS). A large number of measurements can be produced in reasonable time at low costs (Bajorath, 2002; Bleicher *et al.*, 2003; White, 2000). HTS has also been applied to test for synergistic drug combinations (He *et al.*, 2016). In these screens different concentrations of two drugs are applied to a cancer cell line. Despite the importance of cancer cell lines in biomedical research, their ability to accurately represent the *in vivo* state is often questioned. The reason is that even if there is a high genomic correlation between the original tumour and the derived cancer cell line, it is still far from perfect (Ferreira *et al.*, 2013). Furthermore, testing the complete combinatorial space with HTS is unfeasible (Goswami *et al.*, 2015; Morris *et al.*, 2016).

Computational methods such as machine learning models offer the possibility to efficiently explore the large synergistic space. Accurate predictive models can be generated by leveraging the available HTS synergy data. Reliable predictions provide guidance for *in vitro* and *in vivo* research. Furthermore, methods developed to utilize genomic information for their predictions offer the opportunity to apply them also in an *in vivo* setting. Therefore, these predictive models are a big step towards precision medicine (Bulusu *et al.*, 2016). Anti-cancer synergy prediction has already been tackled with a wide variety of approaches. Methods range from systems biology methods (Feala *et al.*, 2010), kinetic models (Sun *et al.*, 2016), mixed integer linear programming methods based on the diseased gene set (Pang *et al.*, 2014), computational methods based on gene expression profiles after treatment with single drugs and dose response curves (Goswami *et al.*, 2015; Yang *et al.*, 2015), to machine learning approaches including Random Forests and Naive Bayes methods (Li *et al.*, 2015; Wildenhain *et al.*, 2015). However, these methods are restricted to certain pathways, targets or cell lines, or require transcriptomic data of cell lines under compound treatment. In contrast, our approach only requires a single transcriptomic characterization of the cell line without compound treatment, and the chemical structure of the two drugs.

Two crowdsourced challenges were focused on computational methods for drug combinations. In 2012, a challenge considered only a single cell line (OCI-LY3) and 14 different drugs (Bansal *et al.*, 2014). Recently, a second challenge was launched to determine the state-of-the-art of prediction of synergy scores (Yu, 2016). The dataset consisted of 3800 synergy score samples of pre-specified drug combinations. Although the research questions addressed in these challenges are undoubtedly of high importance, the competition was grounded on datasets with limited size and did not evaluate the performance of methods for novel drug combinations.

Due to the fast development of high-throughput methods, the amount of available synergy data points has tremendously increased. Publicly available databases such as ASDCD (Chen *et al.*, 2014) containing antifungal combinations and DCDB (Liu *et al.*, 2010, 2014) with approved and investigational combinations, play a key role in providing good quality training data for developing computational predictive methods. A review of these resources has been discussed in Bulusu *et al.* (2016). Recently, a large HTS synergy study (O’Neil *et al.*, 2016) with more than 20 000 synergy measurements was performed, which offers the possibility to evaluate computational methods for predicting novel drug combinations. The dataset covers 38 drugs and 39 cancer cell lines. Therefore, the performed HTS covered 83% of the possible two drug combinations. Both experimental and approved drugs were tested. The used cancer cell lines originated from seven different tissue types.

Previous methods were developed and optimized for small datasets. However, methods developed for limited data might not be appropriate anymore. Predictive performance can be improved, since more data is available and methods which learn from tens of thousands of data points can be used. Deep Neural Networks (DNNs), which strongly profit from large datasets, have impacted many scientific disciplines and achieved new state-of-the-art performance (LeCun *et al.*, 2015). Deep Learning has set new records in image (Farabet *et al.*, 2013; Krizhevsky *et al.*, 2012) and speech recognition (Hinton *et al.*, 2012; Sainath *et al.*, 2013). Recently, DNNs also found their way into drug design (Ma *et al.*, 2015; Mayr *et al.*, 2016). DNNs have the ability to learn abstract representations from high-dimensional data, which is useful for solving complex tasks. The difficult process of identifying new drugs includes many challenges for which Deep Learning is perfectly suited, due to large amounts of available data and its ability to extract important features (Unterthiner *et al.*, 2015).

In this work, we present DeepSynergy, a Deep Learning approach for predicting the synergy of drug combinations and a thorough method comparison. The model was designed for regression, since treating the task as a classification problem might oversimplify the actual situation (Chen *et al.*, 2016; Pahikkala *et al.*, 2015). DeepSynergy uses both compound as well as genomic information as inputs. By incorporating genomic information, DeepSynergy can learn to distinguish different cancer cell lines and find specific drug combinations that have maximal efficacy on a given cell line. DeepSynergy combines information about the cancer cell line and the drug combination in its hidden layers to form a combined representation that eventually leads to accurate predictions of drug synergies. DeepSynergy was trained on a large publicly available synergy dataset (O’Neil *et al.*, 2016). To benchmark the performance of our approach we compare the results to other state-of-the-art machine learning methods: Gradient Boosting Machines (Friedman, 2001), Random Forests (Breiman, 2001), Support Vector Machines (Cortes and Vapnik, 1995) and Elastic Nets (Zou and Hastie, 2005). A median polishing method serves as baseline for the task. Overall, we found that DeepSynergy can predict drug synergies of novel combinations within the space of explored drugs and cell lines with high accuracy and significantly outperforms the other methods at this task. Furthermore, we investigated the performance of the methods at extrapolating to drug combinations which include either novel drugs or novel cell lines and found that these scenarios are challenging for all methods.

## 2 Materials and methods

### 2.1 Dataset

**Fig. 1.**
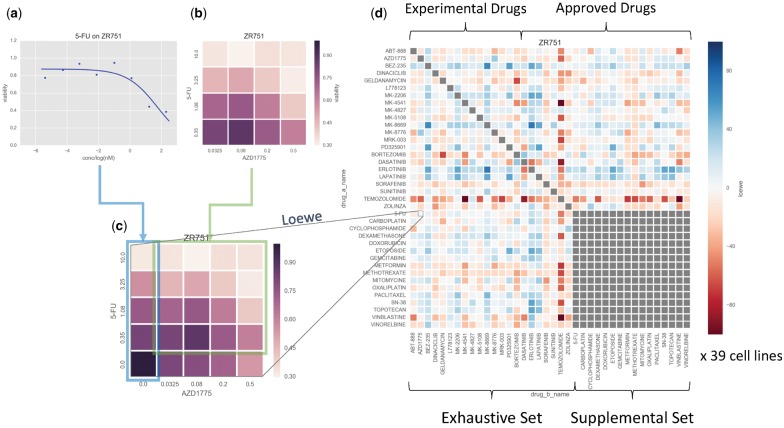
Synergy calculation workflow. (**a**) Single agent screens at 8 concentration points were run for each of the 38 compounds against each of the 39 cell lines. (**b**) Checkerboards of 4-by-4 nonzero concentrations were measured for each of the 583 tested combinations, again for each of the cell lines. (**c**) Values at the checkerboard concentrations were interpolated from the fitted Hill curves from (**a**), and combined with the measured checkerboards from (**b**) to yield a 5-by-5 matrix, from which Loewe synergy values could be obtained. (**d**) The procedure from (**c**) was performed for each pairwise combination of the drug pairs. Notably self–self combinations were not explicitly measured. Furthermore, pairwise combinations within a set of 16 of the drugs (the ‘supplemental’ set) were similarly not measured (hence the gray block in the bottom right of the heatmap). This procedure was repeated for each cell line to yield a 38 × 38 × 39 data cube of input data, from which training, validation and test data were drawn using stratified nested cross-validation

We used a recently published large-scale oncology screen produced by Merck & Co. (O’Neil *et al.*, 2016) to train our models. The dataset comprises 23 062 samples, where each sample consists of two compounds and a cell line. Thereby, the dataset covers 583 distinct combinations, each tested against 39 human cancer cell lines derived from 7 different tissue types (see Supplementary Table S1). Pairwise combinations were constructed from 38 diverse anticancer drugs (14 experimental and 24 approved, see Supplementary Table S2), of which 22 were tested exhaustively in combination (the ‘exhaustive’ set), while the remaining 16 (the ‘supplemental’ set) were tested only in combination with those of the exhaustive set (see Fig. 1d). Each sample was assayed according to a 4-by-4 dosing regimen in quadruple replicate, measuring the rate of cell growth relative to control after 48 h. Notably in contrast to previous combination screening protocols (Griner *et al.*, 2014), separate single agent screens using eight concentrations with six replicates were performed, rather than included in the checkerboard; from these, it was possible to define the edges of the combination surface (where one drug is absent) by interpolating values sampled from fitted Hill curves, leading to 5-by-5 concentration point surfaces for each of the samples (see Fig. 1c).

#### *2.1.1* Synergy score

The degree of synergy indicated in a surface is typically quantified by its deviation from that of one simulated according to a theoretical model, such as Loewe Additivity (Loewe, 1953), Bliss Independence (Bliss, 1939), Highest Single Agent (Tan *et al.*, 2012) or the recent Zero Interaction Potency (Yadav *et al.*, 2015). The original publication (O’Neil *et al.*, 2016) released only the raw surfaces as Supplementary Material. Therefore, we calculated Loewe Additivity values using the batch processing mode of *Combenefit* (Di Veroli *et al.*, 2016). At this stage, replicates were averaged resulting in a set of 22 737 (compound, compound, cell line, synergy value) quartets.

#### *2.1.2* Chemical descriptors and genomic features

To represent the input data in numeric form we used both chemical information from the drugs, and genomic information capturing disease biology. After removing salts, the chemical representations were protonated appropriate for pH 7.4 with *OpenBabel* (O’Boyle *et al.*, 2011). Chemical features were then calculated for both drugs of a drug combination. We calculated three different types of chemical features. Counts of extended connectivity fingerprints with a radius of 6 (ECFP_6) (Rogers and Hahn, 2010) were generated with *jCompoundMapper* (Hinselmann *et al.*, 2011). Additionally, *ChemoPy* (Cao *et al.*, 2013) was used to calculate predefined physico-chemical properties. The set of chemical features was completed by binary toxicophore features based on a set of toxicophores collected from the literature. Toxicophores are substructures which are known to be toxic (Singh *et al.*, 2016). The chemical feature space was reduced by filtering out zero variance features. The final set of chemical features consists of 1309 ECFP_6, 802 physico-chemical and 2276 toxicophore features.

The cell lines were described by their gene expression profile. The profiles of the untreated cells were downloaded from the ArrayExpress database (accession number: E-MTAB-3610) (Iorio *et al.*, 2016). The measurements were performed on an Affymetrix Human Genome U219 array plate. The raw data was quantile normalized and summarized with Factor Analysis for Robust Microarray Summarization (FARMS) (Hochreiter *et al.*, 2006). FARMS additionally provides Informative/Non-Informative calls for each gene (Talloen *et al.*, 2007), which were used to filter the gene expression data to a final set of 3984 genomic features.

### 2.2 Deep learning

**Fig. 2.**
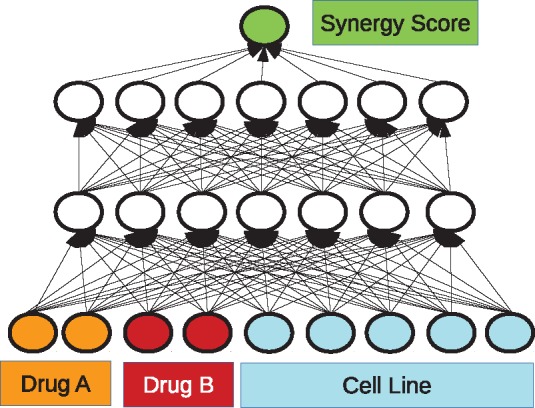
Schematic illustration of our Deep Learning approach. The input consists of three parts: the chemical descriptors for drug A and drug B, and the genomic information of the cell line. The inputs are propagated through the network to the linear output unit. The thereby obtained result is the predicted synergy value. The best performing architecture was determined via exploration of different hyperparameters which are listed in Table 1

DeepSynergy is a feed forward neural network, which maps input vectors representing samples to a single output value, the synergy score. The samples are described by concatenated vectors which include the features of two drugs and one cell line. In Figure 2, the basic setup of DeepSynergy is illustrated. The neurons in the input layer receive the gene expression values of the cell line and chemical descriptors of both drugs as inputs. The information is then propagated through the layers of the DeepSynergy network until the output unit that produces the predicted synergy score. Since the network should not differentiate between the drug combination AB presented in the ordering A-B or B-A, we double the measurements by presenting each sample twice in the training set. Once the drug features are used in an A-B and once in a B-A order. For prediction both ways of sample representation are propagated through the network and averaged. We observed that DeepSynergy learns to predict the same value for drug combination AB in the order A-B and B-A.

We considered different hyperparameter settings, namely different data normalization strategies, in combination with conic or rectangular layers both with different numbers of neurons. Furthermore, we investigated different learning rates as well as regularization techniques. The considered hyperparameter space is summarized in Table 1 and described in more detail in the following.

**Table 1.** Hyperparameter settings considered for DeepSynergy

Hyperparameter	Values considered
Preprocessing	norm; norm+tanh; norm+tanh+norm
Hidden units	[8192, 8192]; [4096, 4096]; [2048, 2048];
	[8192, 4096]; [4096, 2048]; [4096, 4096, 4096];
	[2048, 2048, 2048]; [4096, 2048, 1024];
	[8192, 4096, 2048]
Learning rates	10^−2^; 10^−3^; 10^−4^; 10^−5^
Dropout	no dropout; input: 0.2, hidden: 0.5

*Note*: All possible combinations of the presented hyperparameters were optimized via grid-search.

For data normalization we employed three different types of input normalization: (i) standardizing all inputs to zero mean and unit variance, (ii) standarizing and applying hyperbolic tangent and (iii) standardizing, hyperbolic tangent and standardizing again. The hidden layers apply rectified linear activations (Nair and Hinton, 2010), and the output layer used a linear activation. The mean squared error was the objective function which was minimized. We considered two or three hidden layers with 2048, 4096 and 8192 neurons in the first hidden layer. We tested rectangular layers, that have a constant number of neurons in each hidden layer, and conic layers, in which the number of units halves in each hidden layer. We used stochastic gradient descent with learning rates of 10^−2^, 10^−3^, 10^−4^ and 10^−5^ as optimizer and early-stopping and dropout as regularization techniques. For early-stopping, the adequate number of training iterations was determined by a moving average over 25 epochs on a validation set. For dropout, we either used a dropout rate of 0.2 and 0.5 for the input and the hidden layers, respectively, or no dropout at all. The best hyperparameters were determined using grid search.

### 2.3 Method comparison

We compared DeepSynergy to a baseline method and four state-of-the-art machine learning methods that we adapted to this task. The three different normalization techniques described in the previous section were considered for each method. Furthermore, all methods were allowed to adjust their hyperparameters with grid search. The full range of tested hyperparameters can be found in the Supplementary Material. For all methods their implementation in scikit-learn (Pedregosa *et al.*, 2011) was used.

- **Median Polish.** A median polish model serves as baseline for this task. Since we are not aiming at predicting novel drugs it is possible to make a new prediction based on the median of the two drugs and the cell line involved in the combination. The synergy score is estimated by averaging over the medians of the two drugs and the cell line median.
- **Elastic nets.** Elastic nets (Zou and Hastie, 2005) were used to compare DeepSynergy with a linear method. During hyperparameter selection we considered different values for *α* and the L1 ratio. Supplementary Table S3 summarizes the considered hyperparameter ranges for Elastic Nets.
- **Support Vector Machines (SVMs).** We use a modified version of the MinMax kernel to handle three types of features, binary, counts and continuous ones, simultaneously. This kernel outperformed the Tanimoto and the RBF kernel in previous tasks (Mayr *et al.*, 2016). It is not in the standard implementation of scikit-learn, therefore it was necessary to precompute the similarity of two molecules. The modified MinMax kernel is defined as follows:
  $$\begin{array}{l} K_{\text{modMinMax}}(x,z)=\\ \frac{\sum_{p\in P\;N(p,x)+N(p,z)>0}\frac{\min(N(p,x),N(p,z)}{\max(N(p,x),N(p,z)}}{\sum_{p\in P\;N(p,x)+N(p,z)>0}1} \end{array}$$

  where *N*(*p*, **x**) quantifies feature *p* for compound **x**, and P are the considered features. The kernel matrix is calculated from the preprocessed data, which is then split into a positive and negative part since the kernel function can only be applied to positive values. We used a *ν*-Support Vector Machine for regression and tuned the hyper parameters *ν* and C. Supplementary Table S4 summarizes the considered hyperparameter ranges for *ν*SVMs.

- **Random Forests.** We considered different numbers of estimators (trees) and tuned the number of features considered in each split. Supplementary Table S5 summarizes the considered hyperparameters for Random Forests.
- **Gradient Boosting Machines.** We trained a Gradient Boosting Machine for regression with different numbers of trees, numbers of features considered in each split and different learning rates. Supplementary Table S6 summarizes the considered hyperparameter ranges for Gradient Boosting Machine.

### 2.4 Stratified nested cross-validation

**Fig. 3.**
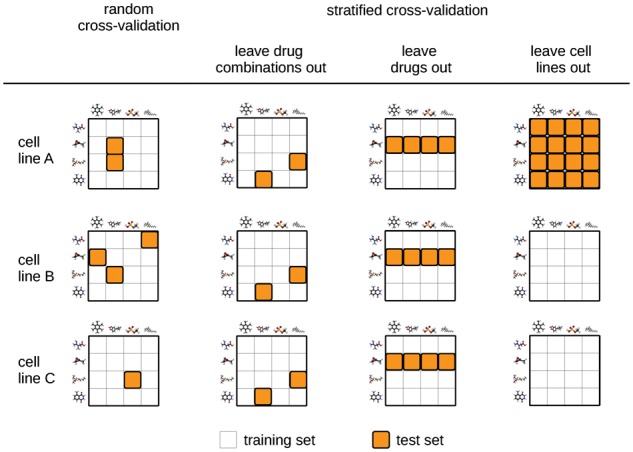
Different cross validation strategies. Random cross validation is shown in the first column. Three different stratified cross validation strategies are shown in the following columns. White and orange squares indicate train set and test set samples, respectively (Color version of this figure is available at *Bioinformatics* online.)

In order to benchmark the performance of DeepSynergy with the competing methods we used stratified nested cross validation. Figure 3 illustrates different cross validation strategies. In contrast to random cross validation, the test samples are not distributed randomly across folds. We used a stratified cross validation approach, where the test sets were selected to leave out drug combinations (see Fig. 3 second column). We used a 5-fold nested cross validation setting (Baumann and Baumann, 2014), in which the hyperparameters are selected in an inner loop based on the validation error. The best performing model of the inner loop was then assessed on an outer test fold to obtain a performance estimate unbiased by hyperparameter selection. Furthermore, we performed stratified cross validation to determine the generalization ability of methods for novel drugs (see Fig. 3 third column) and novel cell lines (see Fig. 3 fourth column).

## 3 Results

### 3.1 Synergy scores

**Fig. 4.**
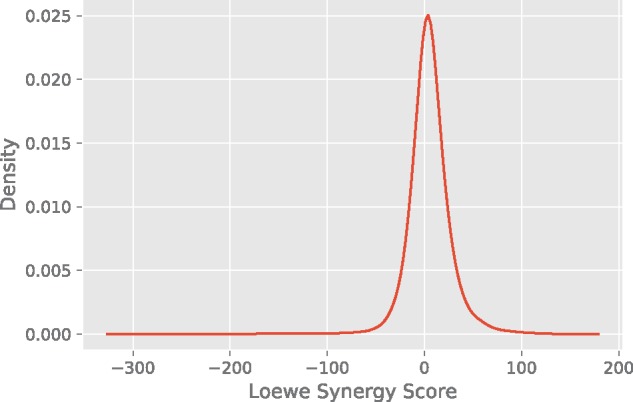
Density plot displaying the distribution of the synergy scores. On the x-axis the synergy scores calculated with Combenefit are shown. On the *y*-axis the density is displayed. Most of the values are close to zero, i.e. additive. High values indicate highly synergistic combinations, whereas combinations with low values exhibit only subadditive effects

Figure 4 displays the density distribution of the synergy scores based on the Loewe model. The values range from −326 to 179. The median and the standard deviation of the distribution are 4.37 and 22.89, respectively. By definition all synergy scores above zero are synergistic. However, drug combinations exhibiting a highly synergistic effect are attractive candidates for clinical studies. Therefore, we focused on the top 10%. Synergistic combinations with a measured score higher than 30 were classified as positive. In the negative class antagonistic, additive and low synergistic drug combinations were summarized.

### 3.2 Method comparison

The methods were compared based on their ability to predict synergy values of novel drug combinations. The primary metric is the mean squared error (MSE), for which the models were optimized for during training. Additionally, we report the mean root mean squared error (RMSE) and the mean Pearson correlation coefficient of each method. Table 2 summarizes the performance of the different methods based on the MSE, RMSE and Pearson correlation coefficient for left out drug combinations respectively.

**Table 2.** Methods comparison based on mean squared error (MSE) with corresponding confidence intervals and *P*-values, mean root mean squared error (RMSE) as well as mean Pearson correlation coefficient over the five test folds

Method	MSE	Confidence Interval	*P*-value	RMSE	Pearson’s *r*
Deep Neural Networks	255.49	[239.93, 271.06]		15.91 ± 1.56	0.73 ± 0.04
Gradient Boosting Machines	275.39	[258.24, 292.54]	9.6 × 10^−17^	16.54 ± 1.37	0.69 ± 0.02
Random Forests	307.56	[286.83, 328.29]	1.2 × 10^−73^	17.49 ± 1.63	0.65 ± 0.03
Support Vector Machines	398.39	[371.22, 425.56]	<10^−280^	19.92 ± 1.28	0.50 ± 0.03
Elastic Nets	420.24	[393.11, 447.38]	<10^−280^	20.46 ± 1.29	0.44 ± 0.03
Baseline (Median Polish)	477.77	[448.68, 506.85]	<10^−280^	21.80 ± 1.49	0.43 ± 0.02

DeepSynergy achieved a test MSE of 255, while Gradient Boosting Machines and Random Forests attained only inferior performance of 275 and 308, respectively. Support Vector Machines and Elastic Nets performed similar with MSEs of 398 and 420, respectively, while median polish, which was used as a baseline, achieved the worst result with an MSE of 478. The relative improvement of the best performing method to the baseline is 53%. A Wilcoxon signed rank-sum test was used to determine if the differences in the mean squared errors are significant and all the *P*-values are below 0.05. Hence, DeepSynergy outperforms the other machine learning methods significantly with regard to MSE, RMSE and Pearson correlation.

Additionally, we evaluated the performance on novel drugs and novel cell lines. The predictive performance of all methods both on novel drugs and novel cell lines is considerably worse than on novel drug combinations. Across methods, the MSEs for the prediction of novel drugs range between 414 and 500, and the MSEs for the prediction of novel cell lines range between 387 and 461 (see Supplementary Section S4). Therefore, the best performing method shows only a relative improvement of 16 and 17% compared to the baseline method that neither uses compound nor cell line features. We hypothesize that the low predictive performance arises from the low number of training examples in terms of chemical compounds (38 examples) and cell lines (39 examples). Therefore, synergy datasets with larger numbers of chemical compounds and cell lines could represent a large boost for predictive synergy models.

### 3.3 Performance metrics

RMSE, MSE depend on the dataset and are therefore difficult to compare across different datasets. To further characterize the predictive performance of DeepSynergy and to give comparable measures, we also provide performance measures that are typical for classification tasks: area under the receiver operator characteristics curve (ROC AUC), area under the precision recall curve (PR AUC), accuracy (ACC), balanced accuracy (BACC), precision (PREC), sensitivity (TPR), specificity (TNR) and Cohen’s Kappa. In Table 3 all performance measures are summarized. When we consider this task as binary classification task with a synergy value threshold of 30, our approach achieves a mean ROC AUC and ROC PR of 0.90 and 0.56, respectively. Since in the training data a lot of additive combinations (i.e. synergy score around 0) are present, the model tends to make more conservative predictions. Therefore, it is important to choose an appropriate threshold for binarising the predicted synergy scores. The threshold for predictions of DeepSynergy was optimized for balanced accuracy on the validation set. With this procedure to select the threshold, DeepSynergy exhibits an ACC, BACC, PREC, TPR, TNR and Kappa of 0.92, 0.76, 0.56, 0.57, 0.95 and 0.51, respectively.

**Table 3.** Performance metrics for the classification task

Performance Metric	ROC AUC	PR AUC	ACC	BACC	PREC	TPR	TNR	Kappa
Deep Neural Networks	**0.90 ± 0.03**	**0.59 ± 0.06**	*0.92 ± 0.03*	*0.76 ± 0.03*	*0.56 ± 0.11*	0.57 ± 0.09	*0.95 ± 0.03*	**0.51 ± 0.04**
Gradient Boosting Machines	*0.89* ± 0.02	*0.59 ± 0.04*	0.87 ± 0.01	**0.80 ± 0.03**	0.38 ± 0.04	**0.71** ± 0.05	0.89 ± 0.01	0.43 ± 0.03
Random Forests	0.87 ± 0.02	0.55 ± 0.04	**0.92** ± 0.01	0.73 ± 0.04	**0.57 ± 0.04**	0.49 ± 0.08	**0.96 ± 0.01**	*0.48 ± 0.04*
Support Vector Machines	0.81 ± 0.04	0.42 ± 0.08	0.76 ± 0.06	0.73 ± 0.03	0.23 ± 0.04	*0.69 ± 0.08*	0.77 ± 0.07	0.24 ± 0.05
Elastic Nets	0.78 ± 0.04	0.34 ± 0.10	0.75 ± 0.05	0.71 ± 0.02	0.21 ± 0.03	0.65 ± 0.07	0.76 ± 0.06	0.22 ± 0.03
Baseline (Median Polish)	0.77 ± 0.04	0.32 ± 0.09	0.76 ± 0.04	0.70 ± 0.03	0.22 ± 0.03	0.62 ± 0.06	0.78 ± 0.04	0.22 ± 0.04

*Note*: All values are mean values ± one standard deviation. The best and second best performance is shown in bold and italic, respectively. The columns provide the performance measures area under ROC curve (ROC AUC), area under precision-recall curve (PR AUC), accuracy (ACC), balanced accuracy (BACC), precision (PREC), sensitivity (TPR), specificity (TNR) and Kappa.

### 3.4 Comparison with previous studies

Our predicted synergy scores agree with observations in previous studies, examples of which are as follows. Kano et al. conducted experiments to evaluate the response of cell line PA-1 to combinations including Paclitaxel. They reported additive effects for Paclitaxel in combination with SN-38 (Kano *et al.*, 1998a), Vinorelbine (Kano *et al.*, 1999b), 5-FU (Kano *et al.*, 1996), Doxorubicin (Akutsu *et al.*, 1995). Our predictions agree with those previous findings, given that the absolute value of the predicted synergy scores is low (with values of 1.48, −13.0, 0.12 and −10.2, respectively). Furthermore, Paclitaxel was combined with Etopside (Kano *et al.*, 1999a) and Methotrexate (Kano *et al.*, 1998b), resulting in additive/antagonistic effects, which agree with our predictions of synergy values of -19.75 and -23.42, respectively. Furthermore, two other experimental studies focused on Irinotecan of which SN-38 is the active metabolite. In a combination with 5-FU on HT-29 an additive effect was observed (Guichard *et al.*, 1997), which agrees with our prediction of −0.57 for 5-FU and SN-38 on HT-29. Furthermore, they investigated the combination Irinotecan with Oxaliplatin on HT-29, HCT-116 and SW-620 (Guichard *et al.*, 2001). The highest effects were observed for HCT-116, whereas SW-620 showed the lowest response. Our predictions for the cell lines HCT-116, HT-29 and SW-620 are 5.82, 1.72 and 0.90 and therefore agree with the experimentally discovered order. Thus, our predictions are confirmed by previous investigations of the respective drug combinations.

### 3.5 DeepSynergy architecture

The architecture of DeepSynergy was determined by the hyperparameter selection procedure, whose results are given in Supplementary Table S7. This procedure identified that tanh normalization, comprising first standardization and then a hyperbolic tangent followed by a second standardization, performed best. Furthermore, DeepSynergy has conic layers. A possible explanation for the fact that conic layers perform well, is their regularizing effect. The lower number of parameters available in the higher layers, which forces the model to generalize by constructing only the most important representations of chemical properties of the input compound combination. Additionally, a large number of units in the first layer (8192) performed better. A smaller learning rate (10^−5^) and dropout regularization were also essential for learning performant networks. Overall, DeepSynergy has a conic architecture with two hidden layers having 8192 neurons in the first and 4096 in the second hidden layer. It uses *tanh* input normalization, has a learning rate of 10^−5^, an input dropout rate of 0.2 and a hidden layer dropout rate of 0.5.

### 3.6 Applicability domain

**Fig. 5.**
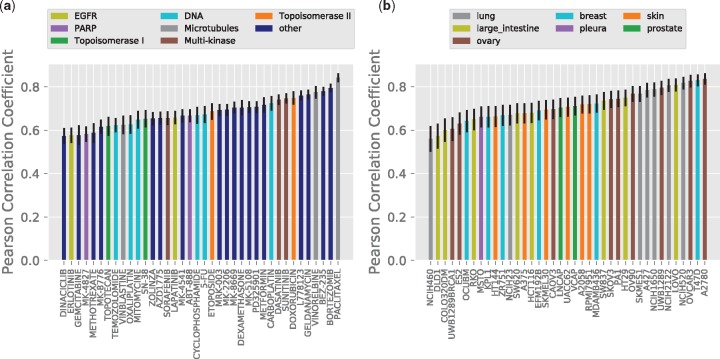
(**a**) Pearson correlation coefficient between true and predicted synergy scores per drugs. On the *x*-axis the drug names are displayed. The bars are colored corresponding to the drug targets. Targets covered only once in the dataset are shown in dark blue. (**b**) Pearson correlation coefficient between true and predicted synergy scores per cell lines. On the *x*-axis the names of the cell lines are displayed. The bars are colored corresponding to the tissues the cell lines originated from. (Color version of this figure is available at *Bioinformatics* online.)

Furthermore, we analyzed performance of DeepSynergy across cell lines and drugs by determining the respective Pearson correlation coefficients. Figure 5 displays the results per (a) drugs and (b) cell lines on the left and right side, respectively.

Correlation coefficients for the set of drugs analyzed range from 0.57 to 0.84. Predicted values for five drugs exhibit a correlation coefficient below 0.6 and 39% of the drugs can be predicted with a correlation coefficient higher than 0.7. On the left side of Figure 5 no clear association between targets and correlation can be observed. Therefore, target specific mechanisms do not explain the performance differences. Furthermore, the performance is not affected by the number of drugs acting on the same target.

Correlation coefficients of the set of cell lines analyzed range from 0.56 to 0.84. Only two cell lines exhibit a correlation below 0.6. More than 50% of the cell lines can be predicted with a correlation higher than 0.7. On the right side of Figure 5 no association can be observed between tissue type and correlation. Nor can performance differences be explained by the number of cell lines originating from a specific tissue type.

We checked whether these performance differences arise from (a) different number of training data points, (b) different quality of synergy scores and (c) different distributions of the synergy scores (see Supplementary Figs S4 and S5), (d) tissue/target specific mechanisms (see Fig. 5a) and (e) tissue/target coverage in the dataset (see Fig. 5b). Neither of these effects showed a clear association with performance. Therefore, we believe that these differences arise (f) from the fact that some biological mechanisms can be modelled better than others. This could be connected to the measurements that we can obtain from cell lines and whether they are able to capture these biological processes. Investigating (f) would require further lab experiments, which is out of scope for this work.

## 4 Availability

We implemented DeepSynergy as a publicly available web-service. The predictions of DeepSynergy are put into context of the training data to improve interpretability of the results. The web-service is available via www.bioinf.jku.at/software/DeepSynergy.

## 5 Discussion

We have developed a novel Deep Learning based method, DeepSynergy, that predicts synergy scores of drug combinations for cancer cell lines with high accuracy. The method requires cancer cell lines to be described by their genomic profiles, and the compounds to be represented by their chemical descriptors. We have demonstrated that DeepSynergy is able to provide best predictions in a cross-validation setting with external test sets, outperforming other methods by a wide margin. Prioritizing drug combinations on the basis of the predictions of DeepSynergy at an AUC of 0.90, could already decrease the time and costs spent on experimental validation (Simm *et al.*, 2018). Since the dataset has only a limited number of different drugs and cell lines all methods show difficulties to generalize well across novel drugs and cell lines. Nonetheless, we are convinced that this limitation can be overcome soon, since dataset sizes increased rapidly over the past years and we expect this trend to continue also in the future. With increased dataset sizes, we presume that our approach can be extended to other areas, in which drug combinations play and important role, for example to antifungals (Chen *et al.*, 2014) and antibiotics (Tamma *et al.*, 2012). With respect to predictive performance, both increased dataset size and algorithmic advances (Klambauer *et al.*, 2017) could further improve DeepSynergy. Overall, our findings suggest that DeepSynergy could be a valuable tool for selecting novel synergistic drug combinations.

## Funding

KP and GK funded by the Institute of Bioinformatics, Johannes Kepler University Linz Austria. KCB and AB thank the European Research Commission (Starting Grant ERC-2013-StG 336159 MIXTURE) for funding.

*Conflict of Interest*: none declared.

## Supplementary Material

Supplementary Data
