# Supplementary material for: DeepSynergy: predicting anti-cancer drug synergy with Deep Learning
Source: Bioinformatics. 2017 Dec 15;34(9):1538–46. doi: 10.1093/bioinformatics/btx806 (PMC5925774; doi:10.1093/bioinformatics/btx806)
Supplement: Supplementary Data [file btx806_supplementarydeepsynergy.pdf]

Publication Supplement
Institute of Bioinformatics, Johannes Kepler University Linz

DeepSynergy: Prediction of anti-cancer drug synergies with Deep Learning
— *Supplementary Information* —

Kristina Preuer [1], Richard P.I. Lewis [2], Sepp Hochreiter [1], Andreas Bender [2], Krishna C. Bulusu [2,3] and Günter Klambauer [1]

[1]Institute of Bioinformatics, JKU, Altenberger Str. 69, 4040 Linz, Austria
[2]Centre for Molecular Science Informatics, Department of Chemistry, University of Cambridge, Lensfield Road, Cambridge CB2 1EW, UK
[3] AstraZeneca, Oncology Innovative Medicines and Early Development, AstraZeneca, Cambridge, UK

S1 Content

This report gives supplementary information to the manuscript "DeepSynergy: Prediction of anticancer drug synergies with Deep Learning". It provides more detailed information in the following three sections: data set, methods and results. The first section describes the drugs and cell lines the data set consisted of. The second section informs about the hyperparamter space for the different methods and about the order independence of Deep Synergy. The last section provides further information for the 3 different cross validation strategies.

S2 Data set

Table S1 displays the cancer cell lines included in the Merck oncology combination screen. The 39 cell lines originated from 7 different tissue types. Table S2 displays the 38 drugs tested in the Merck oncology combination screen. 14 experimental and 24 approved anticancer drugs with diverse targets, modes of action and structure were tested in pairwise combinations against the 39 cell lines. Those in the 'exhaustive' set were combined with all compounds in the set, whereas those in the 'supplemental' set only featured in combination with those in the 'exhaustive' set

cell line	tissue
A2058	SKIN
A2780	OVARY
A375	SKIN
A427	LUNG
CAOV-3	OVARY
COLO-320-DM	LARGE_INTESTINE
DLD-1	LARGE_INTESTINE
EFM-192B	BREAST
ES-2	OVARY
HCT-116	LARGE_INTESTINE
HT-144	SKIN
HT-29	LARGE_INTESTINE
KPL-1	BREAST
LNCAP	PROSTATE
LOVO	LARGE_INTESTINE
MDA-MB-436	BREAST
MSTO-211H	PLEURA
NCI-H1650	LUNG
NCI-H2122	LUNG
NCI-H23	LUNG
NCI-H460	LUNG
NCI-H520	LUNG
OCUB-M	BREAST
OV-90	OVARY
OVCAR-3	OVARY
PA-1	OVARY
RKO	LARGE_INTESTINE
RPMI-7951	SKIN
SK-MEL-30	SKIN
SK-MES-1	LUNG
SK-OV-3	OVARY
SW620	LARGE_INTESTINE
SW837	LARGE_INTESTINE
T47D	BREAST
UACC-62	SKIN
UWB1_289	OVARY
UWB1_289_BRCA1	OVARY
VCAP	PROSTATE
ZR-75-1	BREAST

Table S1: The 39 cell lines tested in the Merck oncology combination screen, covering 7 different tissue types.

compound	target	class	how tested
ABT-888	PARP	experimental	exhaustive
AZD1775	Wee1	experimental	exhaustive
BEZ-235	Phosphatidylinositol-4,5 -bisphosphate 3-kinase	experimental	exhaustive
DINACICLIB	Cyclin-dependent kinases (CDK)	experimental	exhaustive
GELDANAMYCIN	HSP90	experimental	exhaustive
L778123	Farnesyltransferase/ GGPTase-I (FTI/GGTI)	experimental	exhaustive
MK-2206	Protein kinase B (AKT)	experimental	exhaustive
MK-4541	Anti-androgen	experimental	exhaustive
MK-4827	PARP	experimental	exhaustive
MK-5108	Aurora kinase A	experimental	exhaustive
MK-8669	mTOR	experimental	exhaustive
MK-8776	Checkpoint kinase 1 (Chk1)	experimental	exhaustive
MRK-003	γ-secretase	experimental	exhaustive
PD325901	MEK	experimental	exhaustive
BORTEZOMIB	Proteasome	approved	exhaustive
DASATINIB	Multi-kinase	approved	exhaustive
ERLOTINIB	EGFR	approved	exhaustive
LAPATINIB	EGFRs (EGFR/Her2)	approved	exhaustive
SORAFENIB	Multi-kinase	approved	exhaustive
SUNITINIB	Multi-kinase	approved	exhaustive
TEMOZOLOMIDE	DNA	approved	exhaustive
ZOLINZA	Histone deacetylase (HDAC)	approved	exhaustive
5-FU	DNA/RNA	approved	supplemental
CARBOPLATIN	DNA	approved	supplemental
CYCLOPHOSPHAMIDE	DNA	approved	supplemental
DEXAMETHASONE	Glucocorticoid receptor	approved	supplemental
DOXORUBICIN	Topoisomerase II	approved	supplemental
ETOPOSIDE	Topoisomerase II	approved	supplemental
GEMCITABINE	Ribonucleotide reductase	approved	supplemental
METFORMIN	5' AMP activated kinase (AMPK) agonist	approved	supplemental
METHOTREXATE	Dihydrofolate reductase	approved	supplemental
MITOMYCIN	DNA	approved	supplemental
OXALIPLATIN	DNA	approved	supplemental
PACLITAXEL	Microtubules	approved	supplemental
SN-38	Topoisomerase I	approved	supplemental
TOPOTECAN	Topoisomerase I	approved	supplemental
VINBLASTINE	Microtubules	approved	supplemental
VINORELBINE	Microtubules	approved	supplemental

Table S2: The 38 drugs tested in the Merck oncology combination screen.

S3 Methods

Hyperparameters. The hyperparameters of all methods were optimized on the validation set. Tables S3, S4, S5, S6 display the hyperparameters with the corresponding ranges considered for Elastic Nets, Support Vector Machines, Random Forests and Gradient Boosting Trees, respectively. Table S7 displays the 10 best performing hyperparameter settings for Deep Neural Networks on the validation set. Different architectures including conic layers and normal layers of different sizes, learning rates, normalization strategies, dropout or no dropout were considered.

Hyperparameter	Values consided
preprocessing	norm; norm+tanh; norm+tanh+norm;
α	0.1; 1; 10; 100
L1 ratio	0.25; 0.5; 0.75;

Table S3: Hyperparameter space considered for Elastic Nets

Hyperparameter	Values consided
preprocessing	norm; norm+tanh; norm+tanh+norm;
ν	0.05; 0.025; 0.01; 0.005;
C	0.0001; 0.001; 0.01; 1; 100;

Table S4: Hyperparameter space considered for Support Vector Machines

Hyperparameter	Values consided
preprocessing	norm; norm+tanh; norm+tanh+norm;
number of estimators (decision trees)	128; 512; 1024; 2048;
features considered	log2(# of features); sqrt(# of features); 256;

Table S5: Hyperparameter space considered for Random Forests

Hyperparameter	Values consided
preprocessing	norm; norm+tanh; norm+tanh+norm;
number of estimators (decision trees)	128; 512; 1024; 2048;
learning rates	1; 0.1; 0.01;

Table S6: Hyperparameter space considered for Gradient Boosting trees

layers	dropout	input_dropout	learning_rate	norm	validation error
8182_4096_1	0.5	0.2	0.00001	tanh_norm	134.6
8182_8182_1	0.5	0.2	0.00001	tanh_norm	134.9
8182_2048_1	0.5	0.2	0.00001	tanh_norm	134.9
4096_4096_1	0.5	0.2	0.0001	tanh	135.4
2048_2048_1	0.5	0.2	0.0001	tanh	135.5
4096_2048_1	0.5	0.2	0.0001	tanh	135.8
4096_2048_1	0.5	0.2	0.0001	tanh_norm	136.2
8182_8182_8182_1	0.5	0.2	0.00001	tanh_norm	136.8
8182_4096_2048_1	0.5	0.2	0.00001	tanh	137.0
4096_4096_1	0.5	0.2	0.0001	tanh_norm	137.4

Table S7: Performance of hyperparameter settings for Deep Neural Networks.

Order Independence. Drug combinations were presented twice to DeepSynergy in order to generate an order independent network. Both orders (drug A - drug B and drug B - drug A) were used for training and prediction. Therefore, each combination was propagated twice through the network. Figure S1 shows the predictions for the two different ways of ordering. All values are close to the identity line and Pearson correlation coefficient of 0.98 was achieved, which shows that the network is able to neglect the order of the drug combination.

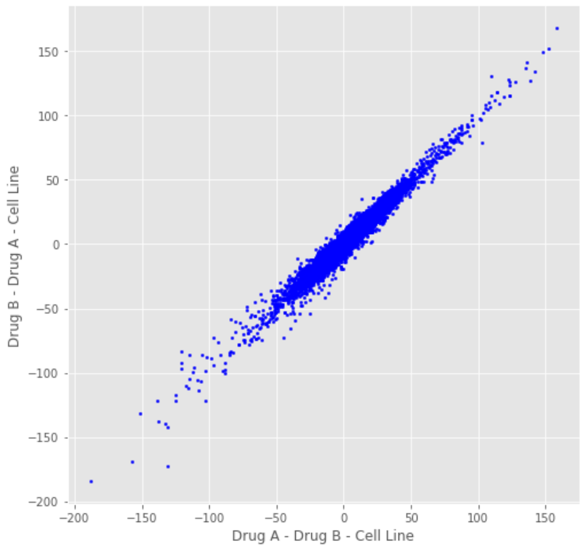

Figure S1: Scatter plot of the predictions obtained by the two different orderings of drug combinations. On the x-axis and y-axis the predictions for the orderings drug A - drug B - cell line and drug B - drug A - cell line are shown, respectively. The Pearson correlation coefficient between the two predictions is 0.98.

S4 Results

Predictive performance on novel drug combinations. In addition to the results shown in the main manuscript we provide the (ROC) and precision recall (PR) curves (Figure S2 and S3), respectively.

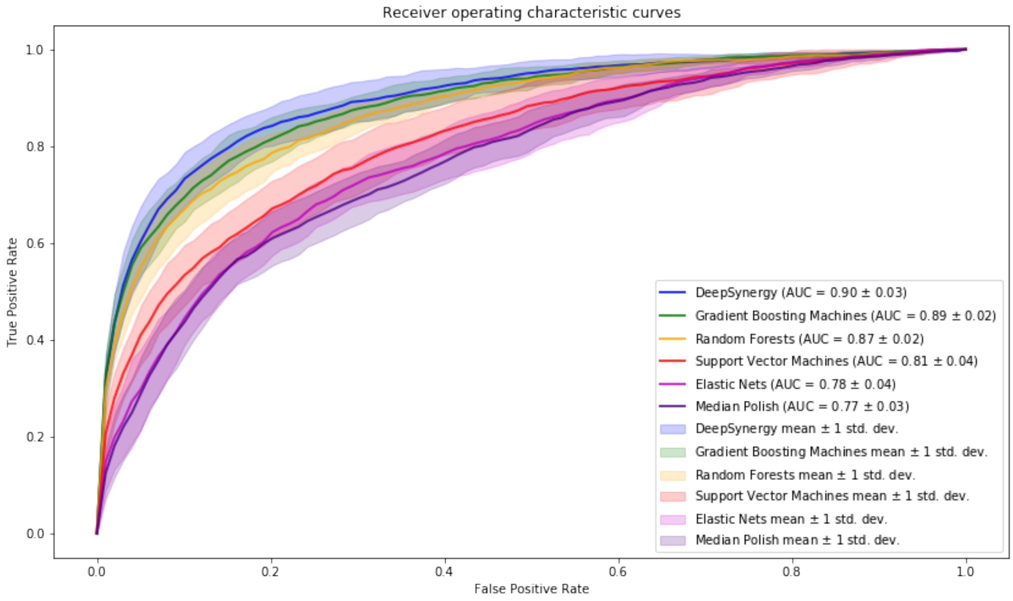

Figure S2: Receiver operating characteristics (ROC) curves for all methods averaged over the 5 cross validation folds. Averaged ROC curves are shown as solid lines. Error bars in terms of one standard deviation are shown as shaded areas. The mean area under curve ± standard deviation is displayed in the legend.

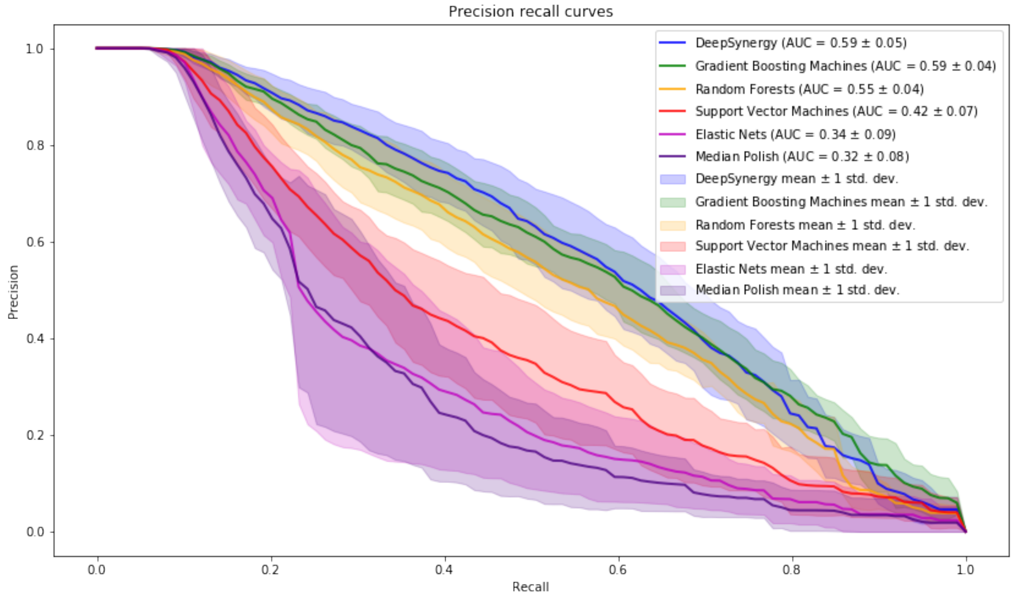

Figure S3: Precision recall (PR) curves for all methods averaged over the 5 cross validation folds. Averaged PRC curves are shown as solid lines. Error bars in terms of one standard deviation are shown as shaded areas. The mean area under curve $\pm$ standard deviation is displayed in the legend.

Measured and predicted synergy scores. Figures S4 and S5 display the distributions of the measured and predicted synergy scores per cell line and drug, respectively. The distributions are ordered by their correlation coefficient between measured and predicted values. Neither the distributions of the predicted nor of the true synergy scores are associated with the performance.

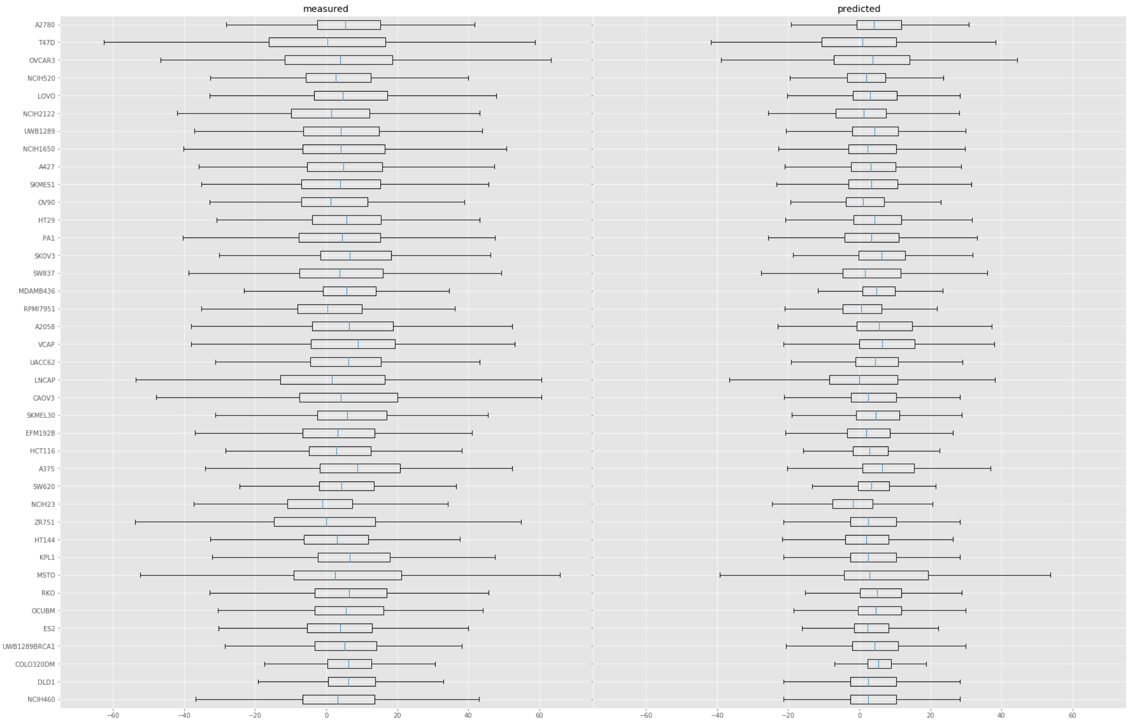

Figure S4: Left: distributions of measured synergy scores per cell line. Right: distributions of predicted synergy scores per cell line. On the x-axis the Loewe synergy score is shown. On the y-axis the cell lines are ordered according to the performance achieved by DeepSynergy. No clear association can be observed.

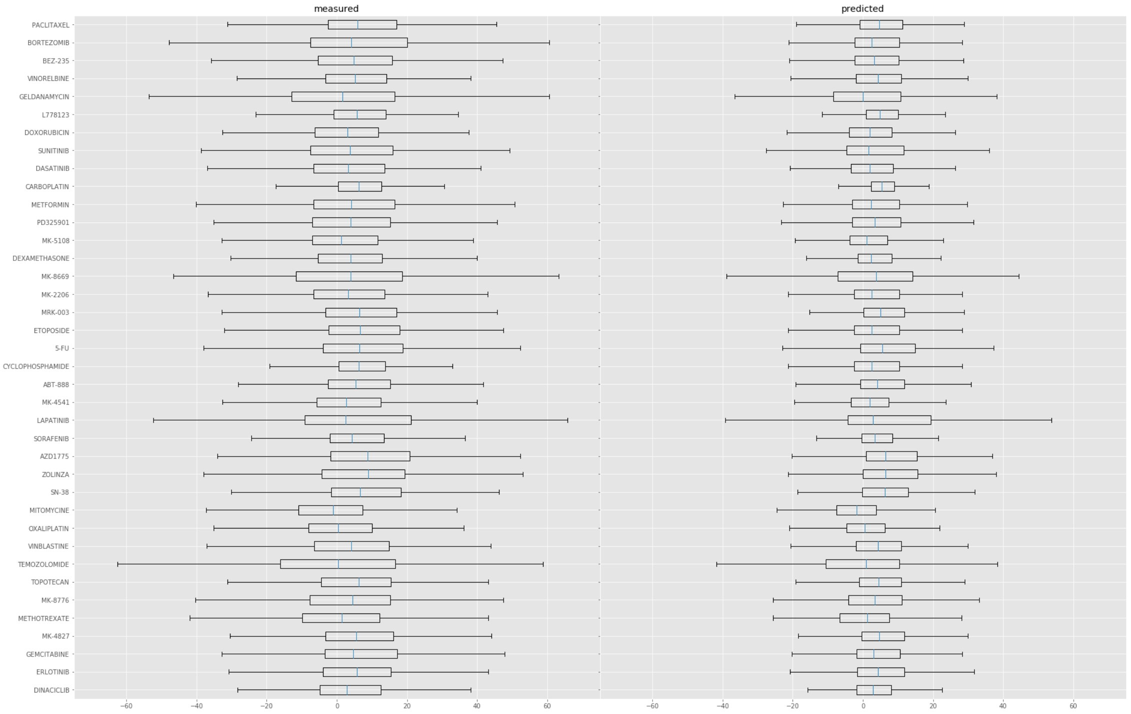

Figure S5: Left: distributions of measured synergy scores per drug. Right: distributions of predicted synergy scores per drug. On the x-axis the Loewe synergy score is shown. On the y-axis the drugs are ordered according to the performance achieved by DeepSynergy. No clear association can be observed.

Predictive performance on novel drugs. We performed a method comparison with respect to the predictive performance on novel drugs, for which we used "leave drugs out" stratified cross validation strategy (see column 3 of Figure 3 in main manuscript) to evaluate the performance. Table S8 shows the methods comparison based on the mean squared error (MSE) with corresponding confidence intervals and p-values. Furthermore, we provide the mean root mean squared error RMSE) and the mean Pearson correlation coefficient over the 38 drugs. Overall, all methods yield a low predictive value and thus do not generalize well enough in order to reliably predict novel drugs. We assume that the low predictive performance is caused by the low number of training examples. Concretely, all models can only be trained on 38 drugs, whereas the space of possible drugs is much larger. In Figure S6 and S7 the methods are compared based on their receiver operating characteristics (ROC) and precision recall (PR) curves obtained on the leave drugs out cross validation, respectively.

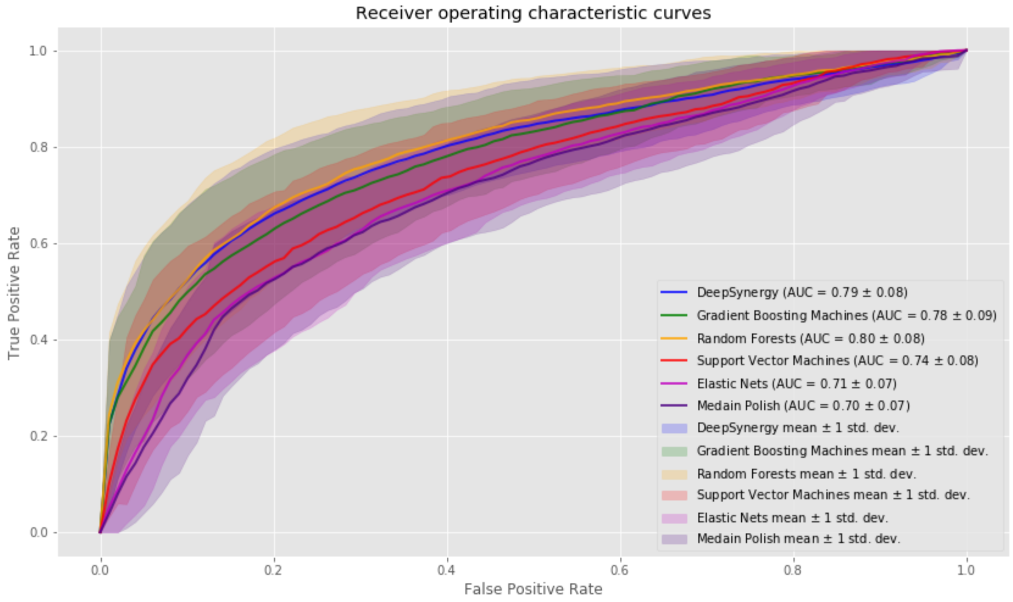

Figure S6: Receiver operating characteristics (ROC) curves for all methods averaged over drugs. Averaged ROC curves are shown as solid lines. Error bars in terms of one standard deviation are shown as shaded areas. The mean area under curve ± standard deviation is displayed in the legend.

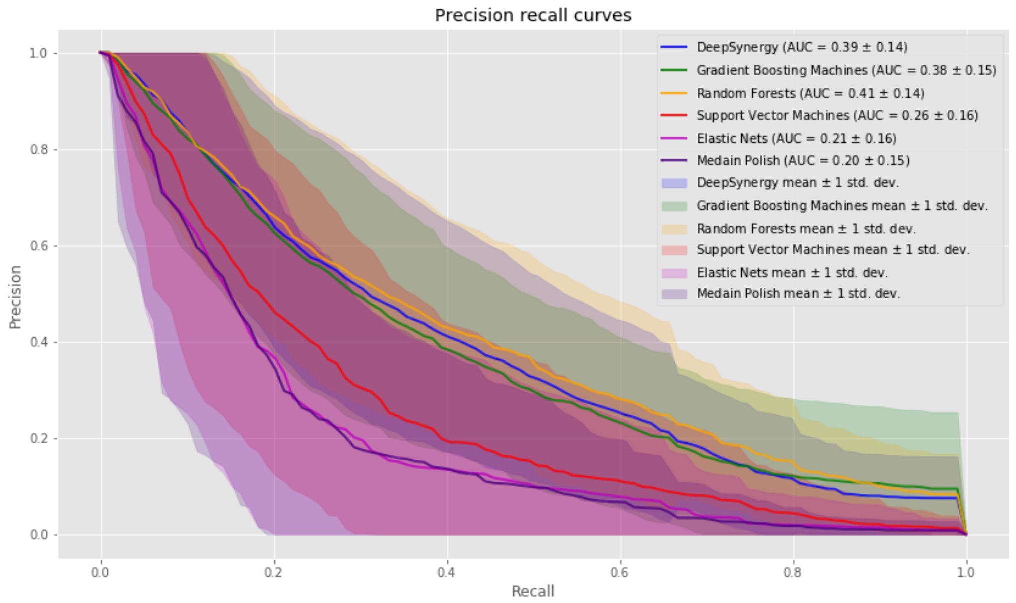

Figure S7: Precision recall (PR) curves for all methods averaged over drugs. Averaged PRC curves are shown as solid lines. Error bars in terms of one standard deviation are shown as shaded areas. The mean area under curve ± standard deviation is displayed in the legend.

Method	Mean Squared Error (MSE)	Confidence Interval	p-value	Root Mean Squared Error (RMSE) ± std	Pearson Correlation ± std
Random Forests	413.51	[396.16, 430.87]		19.41 ± 5.72	0.51 ± 0.09
Deep Neural Networks	435.92	[417.97, 453.87]	3.40^{-20}	19.90 ± 5.60	0.48 ± 0.08
Gradient Boosting Machines	443.93	[426.57, 461.29]	1.13^{-162}	20.21 ± 5.64	0.47 ± 0.10
Support Vector Machines	459.87	[440.10, 479.63]	1.21^{-94}	20.54 ± 5.52	0.39 ± 0.08
Elastic Nets	476.42	[456.54, 496.30]	5.04^{-169}	20.83 ± 5.77	0.35 ± 0.08
Baseline (Median Polish)	499.23	[478.56, 519.89]	1.16^{-212}	21.29 ± 5.95	0.34 ± 0.08

Table S8: Methods comparison for the leave one drug out cross validation based on mean squared error (MSE) with corresponding confidence intervals and p-values, mean root mean squared error (RMSE) as well as mean Pearson correlation coefficient over the 38 drugs.

Predictive performance on novel cell lines. We performed a method comparison with respect to the predictive performance on novel drugs, for which we used “leave cell lines out” stratified cross validation strategy (see column 4 of Figure 3 in main manuscript) to evaluate the performance. Table S9 shows the methods comparison based on the mean squared error (MSE) with corresponding confidence intervals and p-values. Furthermore, we provide the mean root mean squared error (RMSE) and the mean Pearson correlation coefficient over the 39 cell lines. Overall, all methods yield a low predictive value and thus do not generalize well enough in order to reliably predict novel cell lines. We assume that the low predictive performance is caused by the low number of training examples. Concretely, all models can only be trained on 39 cell lines, whereas the space of cancer cell lines is much larger. In Figure S8 and S9 the methods are compared based on their receiver operating characteristics (ROC) and precision recall (PR) curves obtained on the

leave cell lines out cross validation, respectively.

Method	Mean Squared Error (MSE)	Confidence Interval	p-value	Root Mean Squared Error (RMSE) ± std	Pearson Correlation ± std
Random Forests	386.90	[359.18, 414.62]		18.18 ± 7.52	0.59 ± 0.14
Deep Neural Networks	405.40	[377.81, 432.99]	2.02^{-40}	18.74 ± 7.37	0.57 ± 0.14
Gradient Boosting Machines	407.44	[379.42, 435.47]	3.72^{-76}	18.79 ± 7.38	0.57 ± 0.13
Support Vector Machines	422.71	[394.31, 451.10]	2.32^{-78}	19.17 ± 7.42	0.50 ± 0.13
Elastic Nets	435.57	[406.52, 464.62]	1.61^{-115}	19.51 ± 7.41	0.48 ± 0.13
Baseline (Median Polish)	460.69	[431.58, 489.79]	6.27^{-157}	20.18 ± 7.29	0.47 ± 0.13

Table S9: Methods comparison for the leave one cell line out cross validation based on mean squared error (MSE) with corresponding confidence intervals and p-values, mean root mean squared error (RMSE) as well as mean Pearson correlation coefficient over the 39 cell lines.

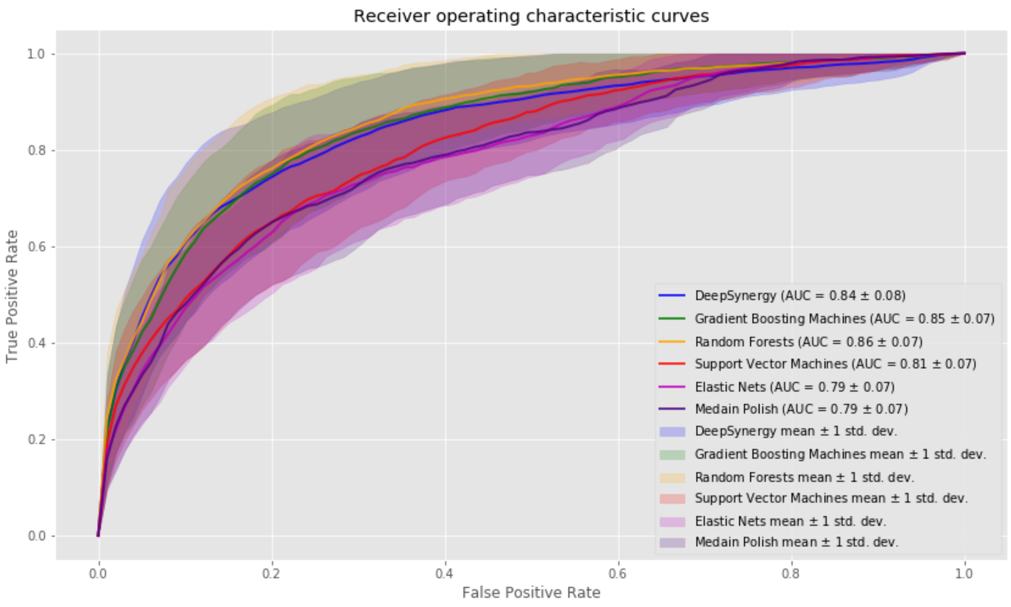

Figure S8: Receiver operating characteristics (ROC) curves for all methods averaged over cell lines. Averaged ROC curves a re shown as solid lines. Error bars in terms of one standard deviation are shown as shaded areas. The mean area under curve ± standard deviation is displayed in the legend.

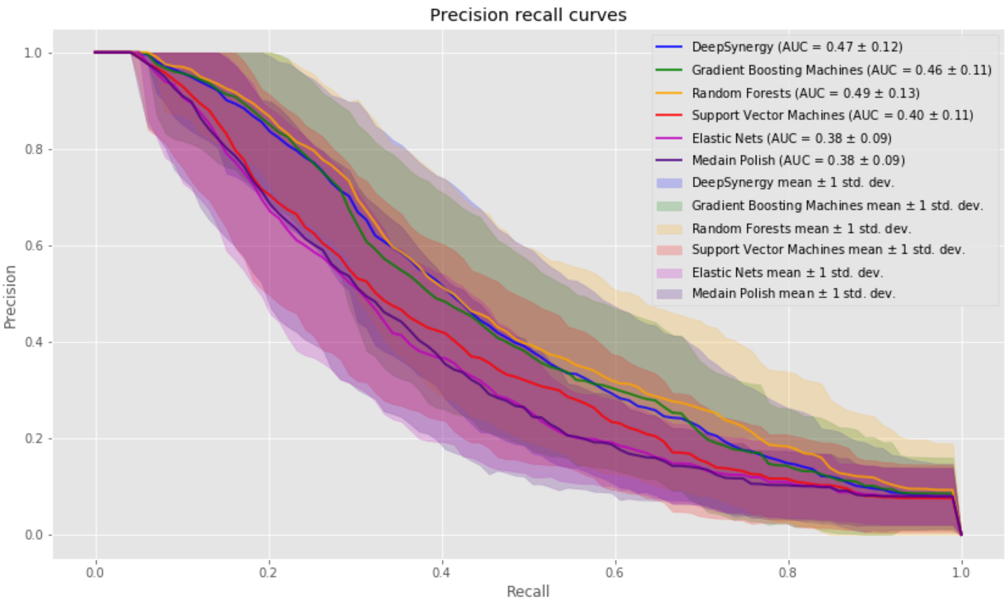

Figure S9: Precision recall (PR) curves for all methods averaged over cell lines. Averaged PRC curves are shown as solid lines. Error bars in terms of one standard deviation are shown as shaded areas. The mean area under curve ± standard deviation is displayed in the legend.
